# Supplementary material for: Gimap5-dependent inactivation of GSK3β is required for CD4+ T cell homeostasis and prevention of immune pathology
Source: Nat Commun. 2018 Jan 30;9:430. doi: 10.1038/s41467-018-02897-7 (PMC5789891; doi:10.1038/s41467-018-02897-7)
Supplement: Supplementary file 1 — Supplementary info [file 41467_2018_2897_MOESM1_ESM.pdf]

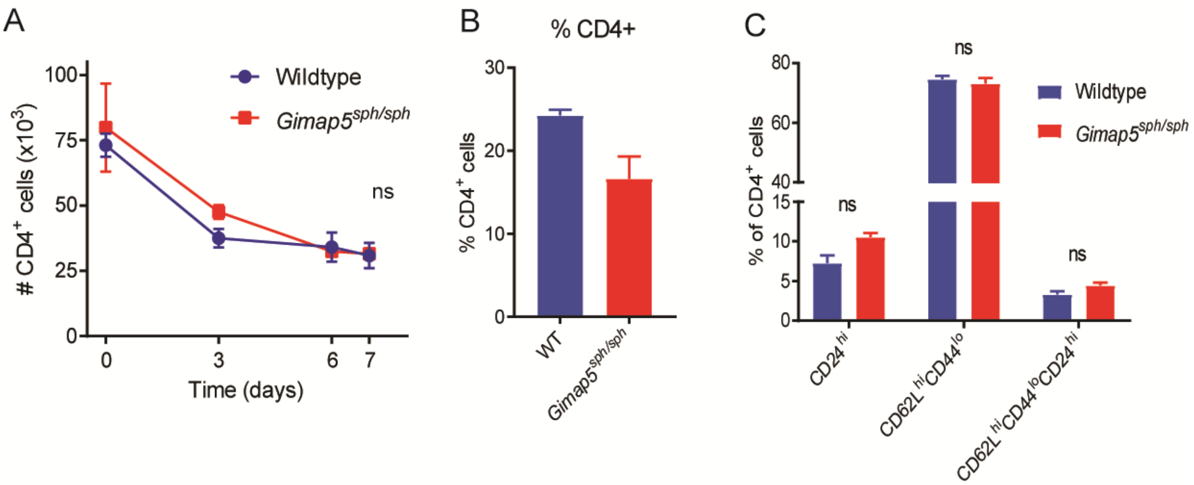

Supplementary Figure 1. Normal thymocyte survival and thymic emigration. (**A**) Number of single positive $CD4^+/CD8^-$ thymocytes surviving *ex vivo* when cultured with IL-7 (5 ng/mL) (*n*=6). Data depict mean ± SD. (**B**) Frequency of peripheral $CD4^+$ T cells (*n*=4). (**C**) Frequency of recent thymic emigrants (RTEs) ($CD24^{hi}$) and naïve ($CD62L^{hi}CD44^{lo}$) cells among peripheral $CD4^+$ T cells from three-week-old mice (*n*=6). Data depict mean ± SEM. Statistical significance is determined by Student's two-tailed test.

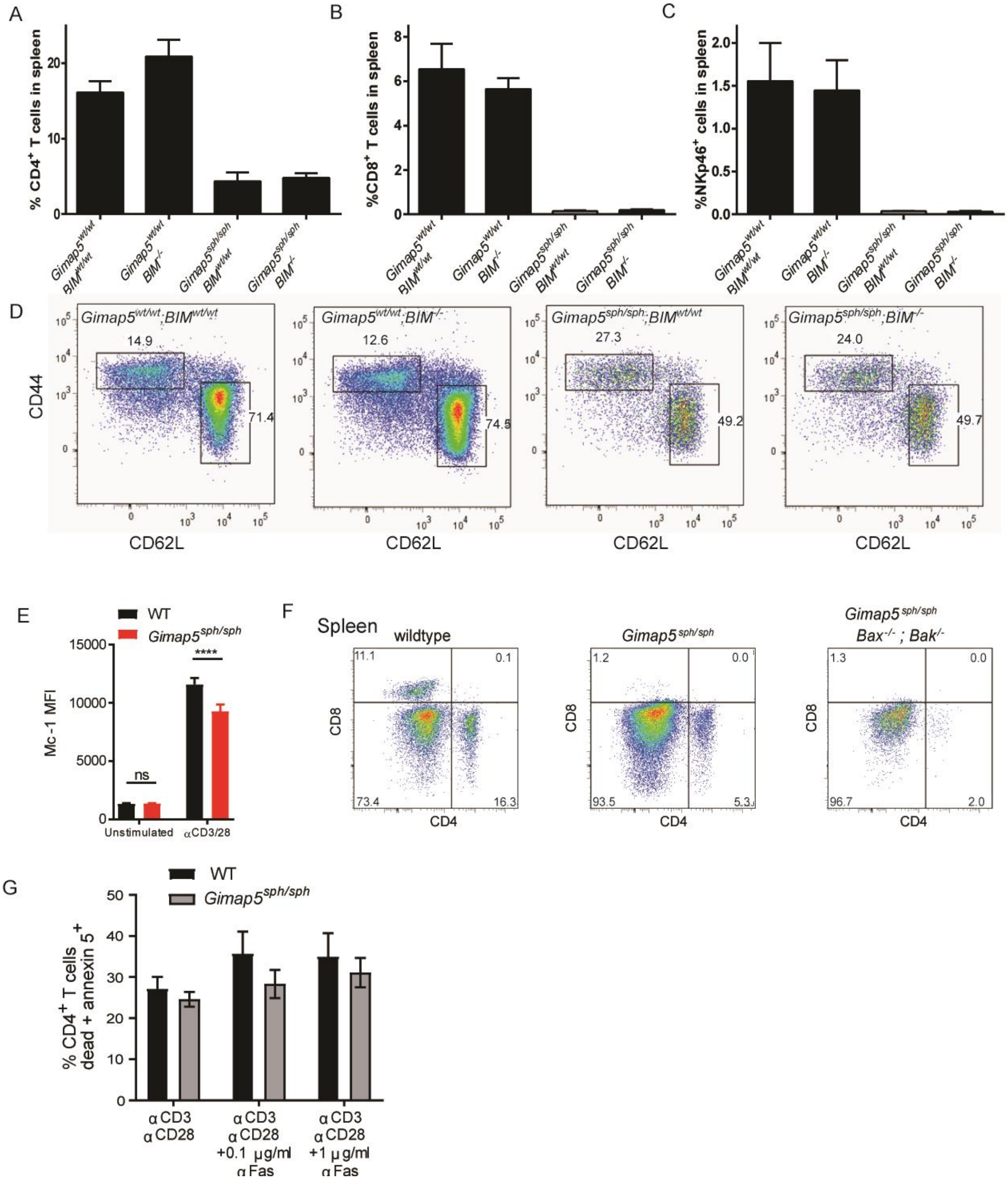

Supplementary Figure 2. Reduced T cell survival is independent of Bim and Bax/Bak. (A-C) Mean frequency ± SD of CD4$^+$ T cells (**A**), CD8$^+$ T cells (**B**), and NK cells (**C**) in the spleen of 5-week-old Bim-deficient *Gimap5$^{sph/sph}$* mice (*n*≥3 mice /group). (**D**) Analysis of CD4$^+$ T cells undergoing lymphopenia-induced proliferation (CD62L^{lo}CD44^{hi}) in the spleen of 5-week-old

Gimap5$^{sph/sph}$ mice deficient for Bim. (**E**) Mcl-1 expression in CD4^{+} T cells stimulated for 24h with αCD3/αCD28 (*n*=4); statistical significance is determined by Student's two-tailed test. (**F**) Frequency of CD4^{+} and CD8^{+} T cells in the spleen of 5-week-old *Gimap5*$^{sph/sph}$ mice lacking pro-apoptotic factors Bax and Bak. Data is representative of two mice/group. (**G**) Frequency of dead + apoptotic CD4^{+} T cells after 8h stimulation with αCD3/αCD28 ± activating αCD95 (anti-Fas). Bars depict mean ± SD (*n*=4).

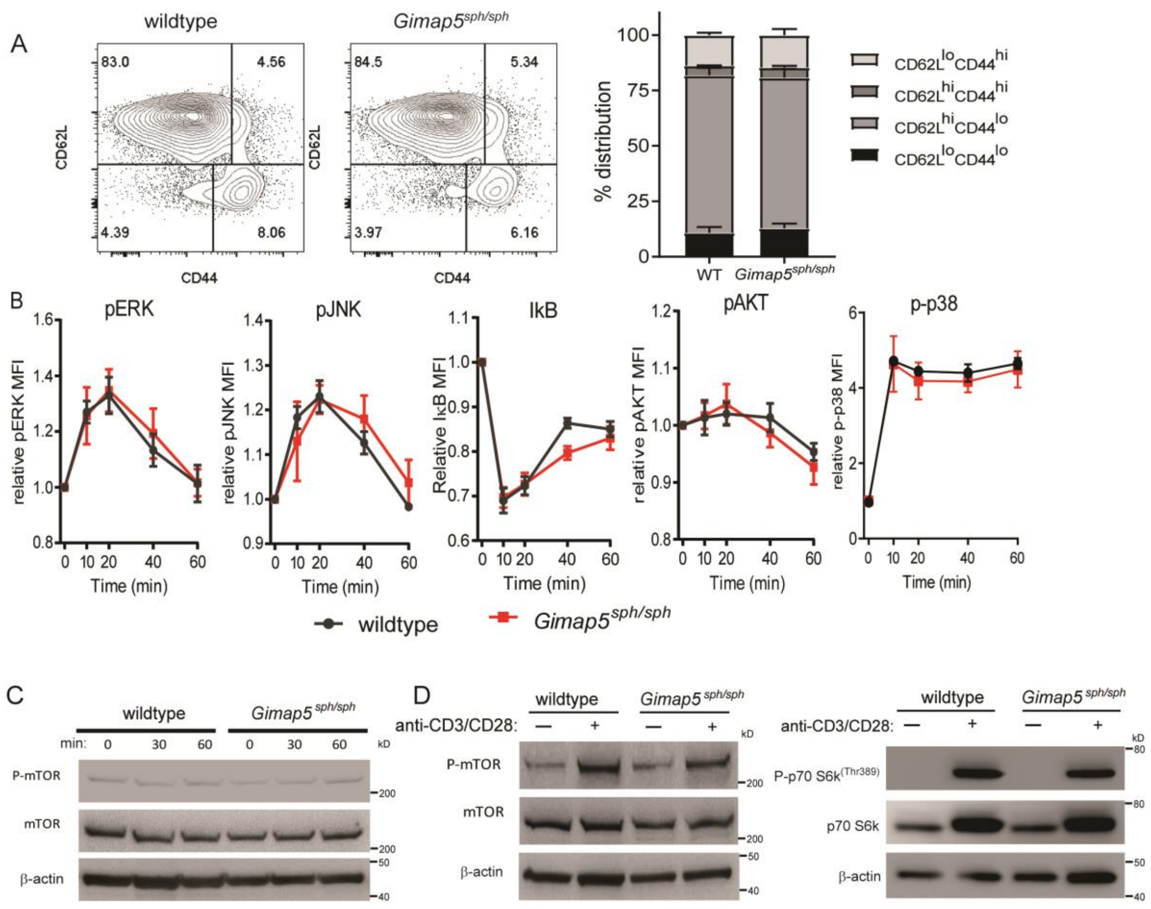

Supplementary Figure 3. Normal proximal CD4^{+} T cell signaling in 3-week-old *Gimap5$^{sph/sph}$* mice. (**A**) Analysis of the percentage of naïve CD4^{+} T cells from 3-week old WT and *Gimap5$^{sph/sph}$* spleens (*n*=4). (**B**) Phosphorylation of ERK (Thr202/Tyr204), JNK (Thr183/Tyr185), AKT (Ser473), and p38 (Thy180/Tyr182) in CD4^{+} T cells from WT and *Gimap5$^{sph/sph}$* mice stimulated with PMA/Ionomycin for the indicated number of minutes. Values measured by flow cytometry and are relative to unstimulated samples and depict mean ± SD (*n*=3). (**C,D**) Immunoblot analyses of mTOR and S6K phosphorylation in CD4^{+} T cells stimulated with αCD3/αCD28 for (**C**) 1h and (**D**) 24h. Immunoblot data depicts representative immunoblots/graphs of at least 3 independent experiments performed on pooled CD4^{+} T cells.

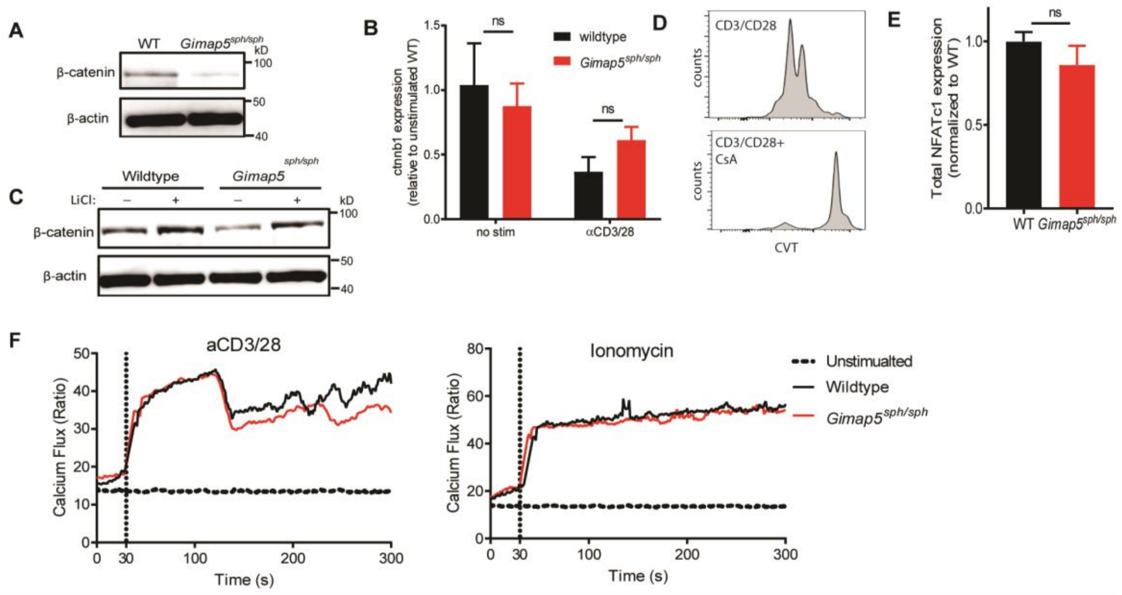

Supplementary Figure 4. Reduced β-catenin but normal NFATc1 expression and calcium flux. Analysis of β-catenin (**A**) protein and (**B**) mRNA in WT and *Gimap5*$^{sph/sph}$ CD4^{+} T cells after 24h of stimulation with αCD3/αCD28 (*n*=6). (**C**) Immunoblot analysis of total β-catenin protein levels after stimulation with αCD3/αCD28 ± 2.5mM LiCl for 24h. Immunoblot data depicts representative immunoblots of at least 3 independent experiments performed on pooled CD4^{+} T cells. (**D**) Proliferation of WT CD4^{+} T cells after 3 days of αCD3/αCD28 stimulation ± calcineurin inhibitor cyclosporin A (200 ng/mL). Plots are representative of 4 independent samples. (**E**) Expression of NFATc1 in WT and *Gimap5*$^{sph/sph}$ CD4^{+} T cells stimulated for 4h with αCD3/αCD28 (*n*=3). (**F**) Representative flow plots detailing calcium flux upon stimulation with αCD3/αCD28 or Ionomycin. Comparison of calcium flux in WT and *Gimap5*$^{sph/sph}$ CD4^{+} T cells upon stimulation with αCD3/αCD28 or Ionomycin. Plots are representative of 4 independent samples. Bars represent mean ± SD. Statistical significance is determined by Student's two-tailed test.

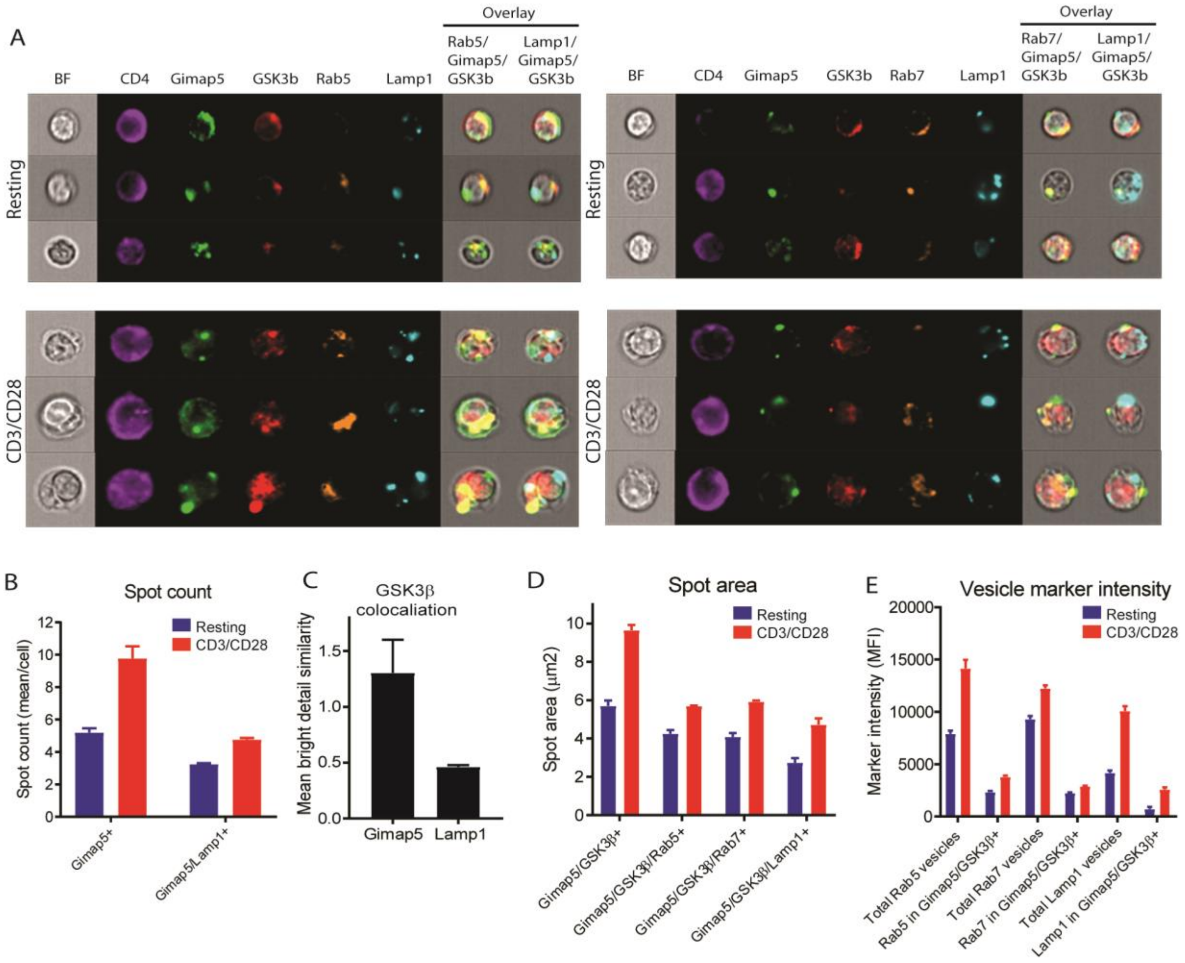

Supplementary figure 5: Vesicular localization of Gimap5 and GSK3β. (**A**) Representative images of WT CD4$^+$ T cells resting or stimulated 24h with αCD3/αCD28. (**B**) Number of Gimap5$^+$ and Gimap5$^+$Lamp1$^+$ vesicles. (**C**) Colocalization of GSK3β with Gimap5 or Lamp1 in activated CD4$^+$ T cells. Colocalization of Gimap5 and GSK3β with Rab5, Rab7, and Lamp1 by (**D**) spot area. (**E**) Intensity of Rab5, Rab7, or Lamp1 within Gimap5$^+$GSK3β$^+$ vesicles. Bars depict mean ± SD (n=4). Each ImageStream data point represents average values of >500 CD4$^+$ T cells. BF: Bright field

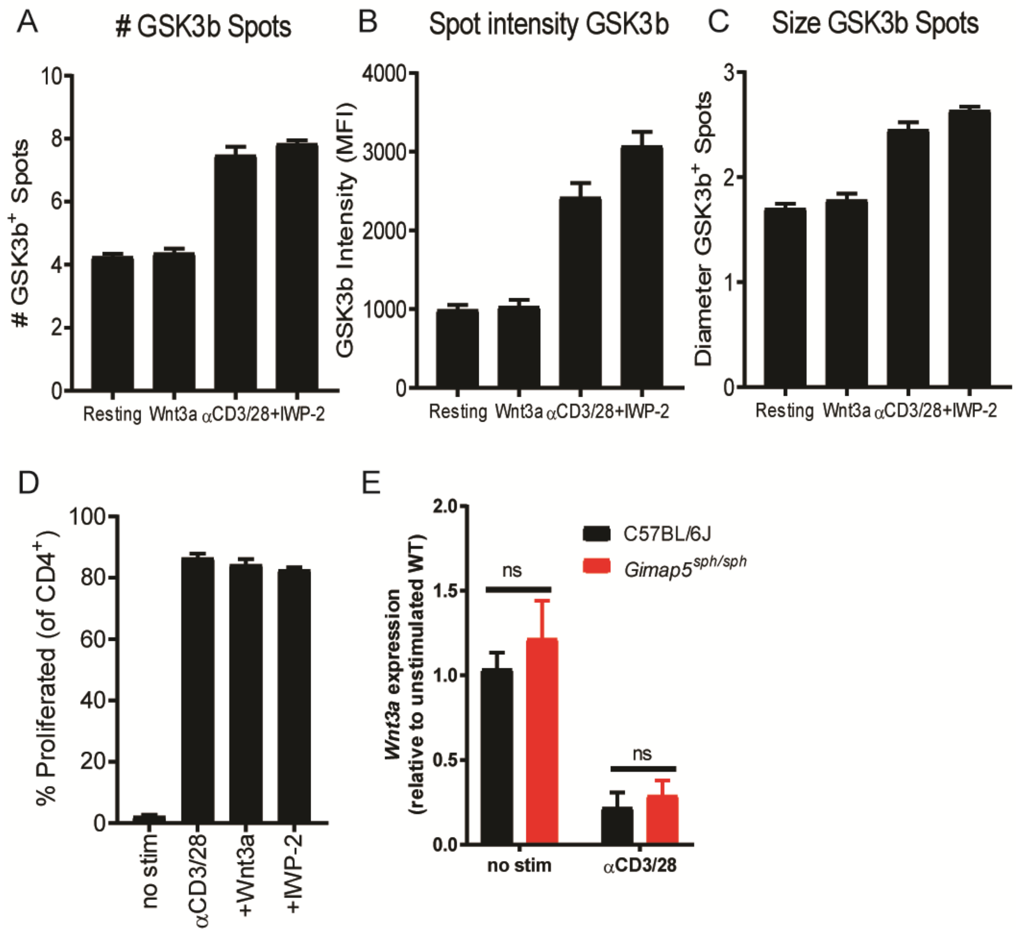

Supplementary figure 6: GSK3β vesicular localization is Wnt independent. (**A-C**) Vesicular localization of GSK3β in WT CD4$^+$ T cells stimulated 24h with 200 ng/mL Wnt3a or αCD3/αCD28 ± 2 µM IWP-2 as measured by (**A**) number of GSK3β$^+$ spots, (**B**) GSK3β vesicular intensity, and (**C**) size of GSK3β$^+$ spots (*n*=3). Each ImageStream data point represents average values of >500 CD4+ T cells. (**D**) Proliferation of WT CD4$^+$ T cells after 3d stimulation with αCD3/αCD28 ± Wnt3a or IWP-2 (*n*=4). Data represent mean ± SD. (**E**) *Wnt3a* mRNA levels in resting and αCD3/αCD28-activated (24h) CD4$^+$ T cells (*n*=9). Bars depict mean ± SEM. Statistical significance is determined by Student's two-tailed test.

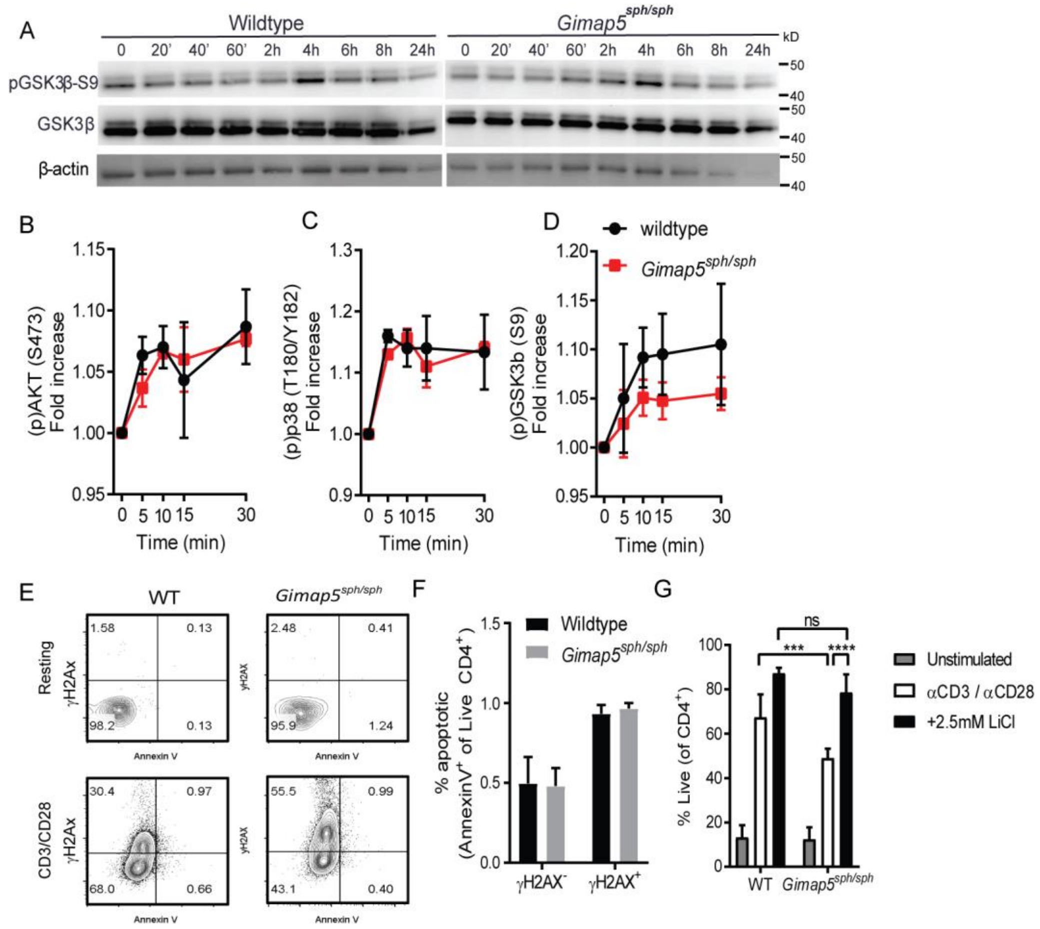

Supplementary Figure 7: **TCR-induced P-Ser^{9} GSK3β is unaffected. (A)** Immunoblot analysis of P-Ser9 GSK3β in WT and *Gimap5$^{sph/sph}$* CD4^{+} T cells during 24 hours activation with αCD3/αCD28. Phosphorylation of (**B**) Akt (S473), (**C**) p38 (T180/Y182), and (**D**) GSK3β (S9) after stimulation with αCD3/αCD28 for the indicated times. Values depicted are relative to unstimulated samples and depict mean ± SD (*n*=5). (**E**) Representative flow plots of live WT and *Gimap5$^{sph/sph}$* CD4^{+} T cells after stimulation with αCD3/αCD28 for 3 days. (**F**) Percent apoptotic (Annexin V^{+}) cells of live stimulated CD4^{+} T cells (*n*=3). (**G**) Survival of WT and *Gimap5$^{sph/sph}$* CD4^{+} T cells after 3d stimulation with αCD3/αCD28 ± 2.5 mM LiCl. Bars represent mean ± SD (*n*=6). Immunoblot data depicts representative immunoblots/graphs of at least 3 independent

experiments performed on pooled $CD4^+$ T cells. Statistical significance is determined by ANOVA followed by Sidak's multiple comparisons test.

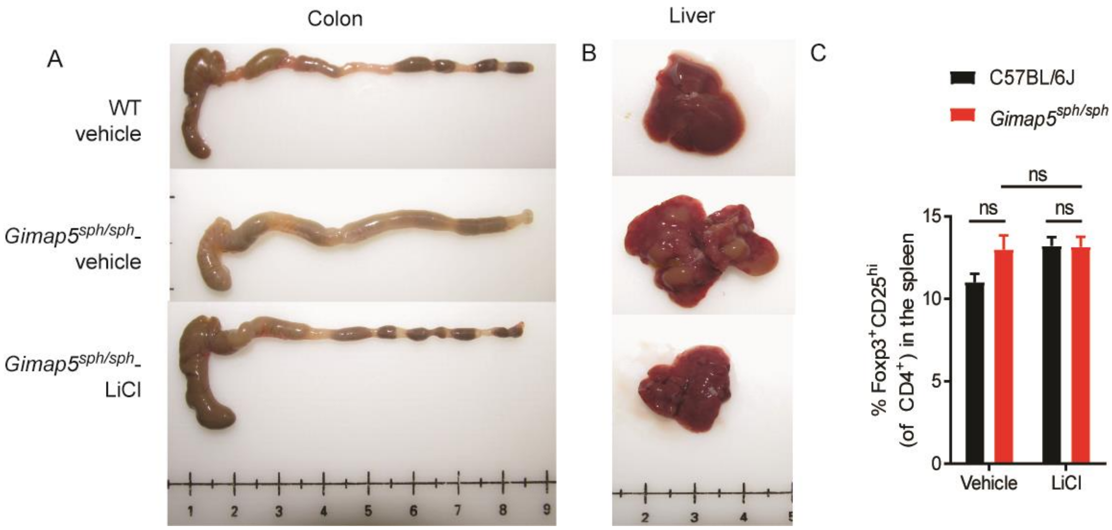

Supplementary Figure 8. Lithium chloride prevents colitis. (**A,B**) Gross morphology of the colon (**A**) and liver (**B**) of Wildtype and *Gimap5*$^{sph/sph}$ mice treated with either LiCl or vehicle from 3 weeks of age to 8 weeks of age. Images representative of 6 mice per group at 7-8 weeks of age. Scale bar shown is in cm. (**C**) Frequency of regulatory T cells ($CD25^{hi}Foxp3^{+}$) within the $CD4^{+}$ T cells population (*n*=6). Bars represent mean ± SD. Statistical significance is determined by ANOVA followed by Sidak's multiple comparisons test.

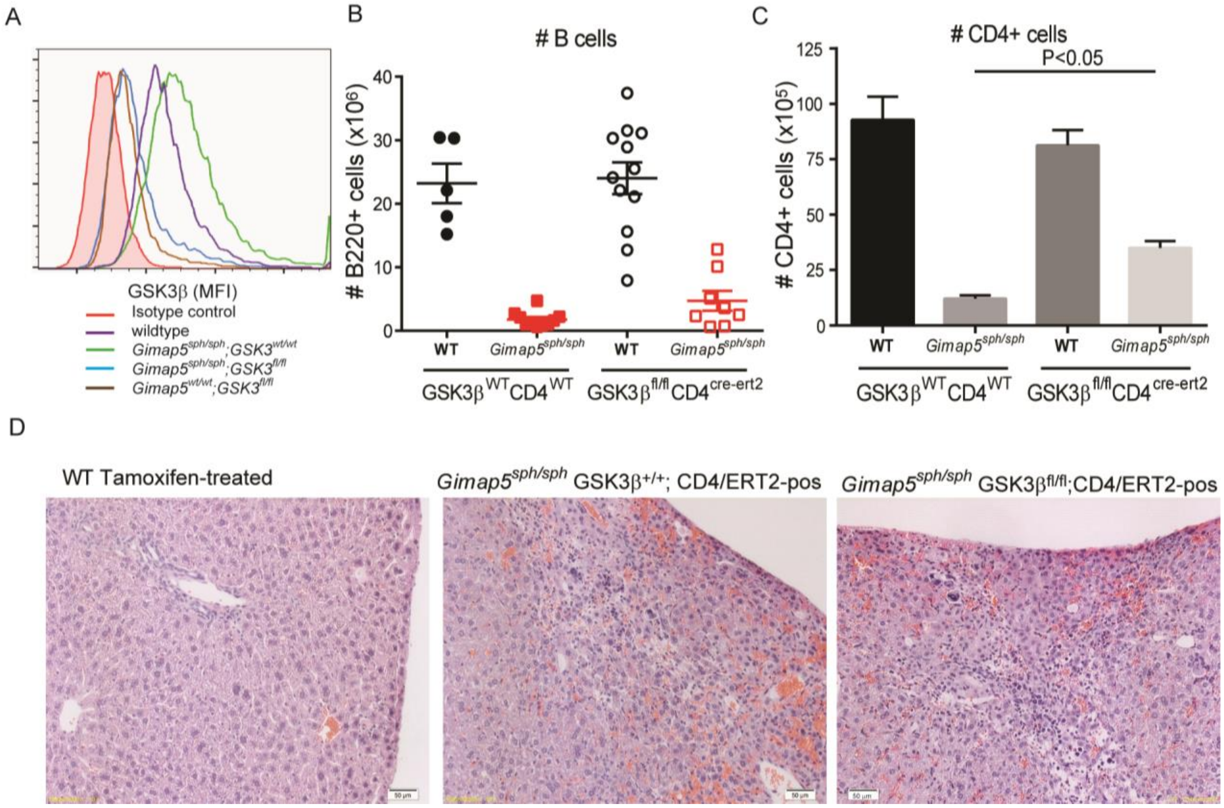

Supplementary Figure 9. Genetic targeting of GSK3β in CD4^{+} T cells improves T cell survival. (**A**) Loss of GSK3β protein in splenic CD4^{+} T cells from *Gsk3β*$^{fl/fl}$*Cd4-cre/ert2* mice treated with tamoxifen from 3 to 8 weeks of age. Representative flow plot is shown. (**B,C**) Absolute number of (**B**) B cells and (**C**) CD4^{+} T cells in the spleen of WT and *Gimap5*$^{sph/sph}$ mice with either GSK3β-sufficient or insufficient CD4^{+} T cells. (**D**) Representative H&E stained liver sections of tamoxifen-treated Wildtype, *Gimap5*$^{sph/sph}$*Gsk3β*$^{WT/WT}$*Cd4-cre/ert2*, and *Gimap5*$^{sph/sph}$*Gsk3β*$^{fl/fl}$*Cd4-cre/ert2* mice at 8 weeks old. Scale bar is 50 µm. Data represent mean values ± SEM from at least 6 mice per group at 8-9 weeks of age; statistical significance is determined by ANOVA followed by Sidak's multiple comparisons test.

Supplementary Table 1. Details of antibodies used.

Antibody	Clone	Supplier	Catalog Number	Dilution
CD4	GK1.5	Biolegend	100437	FC: 1:200<br>ISFC: 1:200
CD44	IM7	BD Pharmigen	564392	FC: 1:200
CD62L	MEL-14	Biolegend	104437	FC: 1:100
CD19	6D5	Biolegend	115531	FC: 1:100
B220	RA3-6B2	ThermoFisher	45-0621-82	FC: 1:100
CD8a	53-6.7	Biolegend	100751	FC: 1:100
NKp46	29A1.4	Biolegend	137617	FC: 1:100
CD25	PC61	Biolegend	102035	FC: 1:100
Foxp3	FJK-165	ThermoFisher	17-5773-82	FC: 1:100
Annexin V	n/a	Biolegend	640906	FC: 1:100
GSK3β	Clone 7/GSK-3b	BD	610202	ISFC: 1:200<br>WB: 1:5000<br>Confocal: 0.7 μg/10^6 cells
pGSK3β (Ser389)	polyclonal	EMD Millipore	07-2275	ISFC: 1:100<br>WB: 1:5000
pGSK3β (Ser9)	D85E12	CST	5558	FC: 1:100<br>WB: 1:1000
Gimap5	MAC421	Barnes *et al., JI* 2010		ISFC: 1:5000<br>Confocal: 1:5000
NFATc1	clone 7A6	Biolegend	649603	ISFC: 1:200
Rab5	polyclonal	abcam	a13253	ISFC: 1:200
Rab7	polyclonal	Sigma	R4779	ISFC: 1:200
Rab11	47/Rab11	BD	610656	ISFC: 1:100
Lamp1	1D4B	Biolegend	121611	ISFC: 1:100
cMyc	D84C12	CST	5605	WB: 1:1000
p-cMyc (T58)	polyclonal	MBS	MBS9405918	WB: 1:1000
p-p53 (S15)	polyclonal	CST	9284	WB: 1:1000
p53	1C12	CST	2524	WB: 1:1000
p-p38 (T180/Y182)	D3F9	CST	4511	FC: 1:400<br>WB: 1:1000
p38	polyclonal	CST	9212	WB: 1:1000
pJNK (T183/Y185)	81E11	CST	4668	FC: 1:100
IκB	polyclonal	CST	9242	FC: 1:100
pERK (T202/Y204)	D13.14.4E	CST	4370	FC: 1:800
pAKT (S473)	D9E	CST	4060	FC: 1:100
p-mTOR (S2448)	D9C2	CST	5536	WB: 1:1000
mTOR	7C10	CST	2983	WB: 1:1000
p-p70 S6K (T389)	108D2	CST	9234	WB: 1:1000
p70 S6K	polyclonal	CST	9202	WB: 1:1000

β-catenin	polyclonal	CST	9562	WB: 1:1000
β-actin	polyclonal	CST	4967	WB: 1:1000
γH2AX	2F3	Biolegend	613403	FC: 1:100<br>ISFC: 1:100
CD4 (human)	OKT4	Biolegend	317444	FC: 2.5 µl/test<br>ISFC: 2.5 µl/test
CD8 (human)	RPA-T8	Biolegend	301005	FC: 20 µl/test
CD3 (human)	OKT3	Biolegend	317337	5 µl/test
GIMAP5 (human)	polyclonal	CST	14108	WB: : 1:1000<br>ISFC: 1:100
CD107b	H4B4	Miltenyi Biotech	130-103-896	ISFC: 1:20
Rabbit IgG	polyclonal	Invitrogen	A-11008	ISFC: 1:200
Rat IgG	polyclonal	Jackson	112-545-167	ISFC: 1:200<br>Confocal: 1:200
Mouse IgG1	polyclonal	Jackson	115-605-205	FC: 1:200<br>ISFC: 1:200<br>Confocal: 1:100
Mouse IgG2a	polyclonal	Jackson	115-065-206	ISFC: 1:2000
Rabbit IgG (HRP)	polyclonal	CST	7074	WB: 1:1000
Mouse IgG (HRP)	polyclonal	CST	7076	WB: 1:1000
Biotin (HRP)	polyclonal	CST	7075	WB: 1:4000

*ISFC: ImageStream Flow Cytometry
**FC: Flow Cytometry
***WB: Western Blot

Supplementary Note 1: Clinical profile description of patient carrying a missense mutation in *GIMAP5* resulting in a Leu204Pro amino acid change

A 14-year-old Caucasian male presented with immune thrombocytopenic purpura (ITP). The patient initially responded to intravenous gammaglobulin (IVIG), however, the thrombocytopenia re-occurred, concomitantly with mild hemolytic anemia, as well as neutropenia and lymphopenia. The patient was positive for a direct Coombs test, but negative for anti-neutrophil or anti-platelet antibodies. A bone marrow biopsy demonstrated mild hypocellularity with low myeloid to erythroid ratio.

Past medical history includes chickenpox infection following varicella vaccine at the age of 11, and shingles at the age of 14. He has suffered and continuous to suffer from extensive warts since the age of 12, which requires frequent cryotherapy. Otherwise, there is no history of recurrent bacterial or fungal infections, enteropathy, lymphadenopathy or other non-hematological autoimmune features.

His immune evaluation prior to referral included normal serum levels of IgG, IgA, IgM, and IgG subgroups, but elevated IgE. He was found to have lymphopenia, with CD4 of 331, CD8 of 67, CD19 of 67, and CD16/56 of 67 cells/mcL. Anti-varicella zoster virus (VZV) antibodies were positive.

At the age 15 he developed a new episode of thrombocytopenia and was given prednisone and IVIG to which he initially responded well, but subsequently became prednisone dependent.

At the age of 16, the patient was referred to our institution for a second opinion for steroid-dependent persistent thrombocytopenia, lymphopenia, and splenomegaly as part of a suspected immunodeficiency disorder. Immune evaluation at the time of referral demonstrated normal Hb/Hct, absolute neutrophil count (ANC: 3120 cells/mcL) and lymphopenia (ALC: 440

cells/mcL) with thrombocytopenia (PLT: 54 K/mcL). Direct Coombs was negative, as were anti-neutrophil and anti-platelet antibodies.

Lymphocyte subset revealed CD3: 273 (62%), CD4: 246 (56%), CD8: 30 (7%), CD19: 135 (31%), CD16/56: 20 (7%) cells/mcL. CD4 to CD8 ratio was 8.2. Serum IgG, A, E levels and IgG subgroups were normal, except low IgM (IgG: 839, IgA: 70, IgM: 54 mg/dl, IgE: 282 IU/ml). Titers to protein antigens were normal and 2/14 pneumococcal titers were protective. B cell panel demonstrated normal proportion of naïve, transitional, and isotype switched $CD27^+$ memory B cells.

He was found to have decreased naive CD4 cells (11.9, N: 33-73.5% for age) with a normal proportion of $CD31^+$ T cells (indicative of recent thymic emigrants), increased memory CD4 cells (88.1, N: 26.3-66.3%), and increased CD4 T-effector memory cells (80.2, N: 33.4-74.1%). Naïve CD8 cells were normal and CD8 TEMRA cells were slightly increased (11.2, N:0-10.3%). The proportion of TCRαβ expression was slightly low on CD8 cells.

T-cell proliferation studies showed reduced proliferation to phytohemagglutinin (PHA) and *Candida*, and borderline response to tetanus. Telomere length measurement revealed low telomere length of total lymphocytes, granulocytes, naïve and memory T cells, but normal telomere length of B cells and NK cells. The patient was given a working diagnosis of immunodeficiency with features of autoimmunity. Mutations in known SCID genes, as well as *LRBA*, *PI3KCD, CTLA-4,* and *STAT3* genes were excluded. Whole exome sequencing revealed a homozygous c.611T>C (p.Leu204Pro) variant in *GIMAP5*, where his parents were found to be heterozygous.
